# Caspase-6 mediates resistance against *Burkholderia pseudomallei* infection and influences the expression of detrimental cytokines

**DOI:** 10.1371/journal.pone.0180203

**Published:** 2017-07-07

**Authors:** Alexander Bartel, André Göhler, Verena Hopf, Katrin Breitbach

**Affiliations:** Friedrich Loeffler Institute of Medical Microbiology, University Medicine Greifswald, Greifswald, Germany; University of Toledo College of Medicine and Life Sciences, UNITED STATES

## Abstract

Caspase-6 is a member of the executioner caspases and known to play a role in innate and adaptive immune processes. However, its role in infectious diseases has rarely been addressed yet. We here examined the impact of caspase-6 in an *in vivo* infection model using the Gram-negative rod *Burkholderia pseudomallei*, causing the infectious disease melioidosis that is endemic in tropical and subtropical areas around the world. *Caspase-6*^-/-^ and C57BL/6 wild type mice were challenged with *B*. *pseudomallei* for comparing mortality, bacterial burden and inflammatory cytokine expression. Bone-marrow derived macrophages were used to analyse the bactericidal activity in absence of caspase-6. Caspase-6 deficiency was associated with higher mortality and bacterial burden *in vivo* after *B*. *pseudomallei* infection. The bactericidal activity of *caspase-6*^-/-^ macrophages was impaired compared to wild type cells. *Caspase-6*^-/-^ mice showed higher expression of the IL-1β gene, known to be detrimental in murine melioidosis. Expression of the IL-10 gene was also increased in *caspase-6*^-/-^ mice as early as 6 hours after infection. Treatment with exogenous IL-10 rendered mice more susceptible against *B*. *pseudomallei* challenge. Thus, caspase-6 seems to play a crucial role for determining resistance against the causative agent of melioidosis. To our knowledge this is the first report showing that caspase-6 is crucial for mediating resistance in an *in vivo* infection model. Caspase-6 influences the expression of detrimental cytokines and therefore seems to be important for achieving a well-balanced immune response that contributes for an efficient elimination of the pathogen.

## Introduction

Caspases are a family of cysteine proteases that are involved in the induction of various forms of cell death and also play a role in inflammatory processes [1,2]. Caspase-6 is a member of the apoptotic effector or executioner caspases, like caspase-3 and caspase-7, but little is yet known about its activation and exact function during apoptosis. The role of caspase-6 has been intensively studied in the pathogenesis of neurodegenerative diseases such as Alzheimer's disease and Huntington’s disease [3,4]. In this context caspase-6 dependent mechanisms were shown to contribute for the exacerbation of neuronal lesions during Huntington’s disease [5] and are critical during the axonal degeneration in the pathogenesis of Alzheimer's disease [3].

In recent years it became evident that caspase-6 plays a pivotal role in inflammatory processes. A previous report could show that caspase-6 cleaves the receptor-interacting protein kinase-1 and it was therefore suggested that caspase-6 might be important for controlling inflammatory events in dying cells [6]. Furthermore it was reported that caspase-6 plays a role for neutrophil-driven activation of macrophages due to the cleavage of IL-1 receptor associated kinase M [7]. In addition, the authors of this study demonstrated that *caspase-6*^-/-^ mice were protected to some degree during cecal lesion induced peritonitis and sepsis [7]. To our knowledge, this is the only report that has been addressing the role for caspase-6 in an *in vivo* infection model yet.

Whereas the knowledge about the role of caspase-6 in infectious diseases is very limited, the impact of the pro-inflammatory caspase-1, a component of the inflammasome [8], has been intensively studied in various infection models [9–12]. Our group could previously show that caspase-1/-11 was essential for mediating resistance against the Gram-negative rod *Burkholderia pseudomallei* in mice [13,14]. *B*. *pseudomallei* is a facultative intracellular bacterium and the causative agent of melioidosis [15]. The infection can be acquired from the surrounding environment in regions in that the soil saprophyte is endemic [16,17]. We could previously demonstrate that macrophages are essential for conferring resistance against *B*. *pseudomallei* in mice [18], but macrophages lacking caspase-1/-11 were not able to control the growth of intracellular *B*. *pseudomallei* [13]. It is known that the killing activity of macrophages as well as *in vivo* resistance against melioidosis is highly dependent on the expression of protective cytokines such as IL-18 and IFN-γ [18–21]. Caspase-1 is a key factor that influences the activation of the pro-inflammatory cytokines IL-18 and IL-1β, a possible explanation for its pivotal role in achieving efficient immunity against melioidosis. In this context the protective effect of IL-18 is mainly associated with the induction of IFN-γ, an essential component for resistance against *B*. *pseudomallei* challenge [18,20]. However, a previous report could demonstrate that IL-1β, which is also activated via caspase-1, has rather detrimental effects during *B*. *pseudomallei* infection [22]. It is considered that IL-1β has inhibitory effects on IFN-γ production and might exhibit direct damaging effects on the host tissue as well [22].

Although there is vivid activity in exploring the role of caspase-1 in *B*. *pseudomallei* and other infection models, the *in vivo* relevance for other caspases have been less well studied so far. In the present report we examined the role of caspase-6 during murine *B*. *pseudomallei* infection and provide evidence that caspase-6 is crucial for determining resistance against this facultative intracellular bacterium. In this context we show that caspase-6 influences the expression of detrimental cytokines that are likely to contribute for the increased susceptibility of mice lacking caspase-6 after *B*. *pseudomallei* challenge.

## Materials and methods

### Bacteria

*Burkholderia pseudomallei* strain E8 is a soil isolate from Thailand. Prior to the experiments, bacteria were either cultured in Luria Bertani broth or on blood agar plates and adjusted to the desired concentration in PBS or respective cell culture medium. The 50% lethal dose for *B*. *pseudomallei* strain E8 at four weeks after i.v. infection was in the rage of 5 x 10^4^–5 x 10^5^ CFU. A dose control was conducted for each infection experiment to evaluate the effective bacterial load that was applied to animals or cells. The respective dose ranges from pooled data are stated in each legend of the figures.

### Animals

Breeding pairs of C57BL/6 *caspase-6*^*-/-*^ mice were kindly provided by Edward A. Clark (Yale University of School of Medicine, USA). Breeding pairs of C57BL/6 control animals were purchased from Charles River (Sulzfeld, Germany). We used six to 10 week old age-matched female animals in all in vivo infection experiments. Animals were bred under specific pathogen-free conditions at MICROMUN (Greifswald, Germany). Our housing facility consists of specific pathogen-free Sealed Lid Individual Ventilated Cages from Tecniplast [Sealsafe Plus Mouse (Green Line)] racks. All experiments were performed under Biosafety level 3 conditions with ISOcage N cages with constant negative air pressure for Biocontainment (Tecniplast, Hohenpeissenberg, Germany). For all cages we used enrichment items (nesting material and plastic igloos), laboratory animal bedding (GOLDSPAN® lab Grade 5, Brandenburg Group, Goldenstedt, Germany). The animals were fed ad libitum (Ssniff, V1535-000, R/M-H). Euthanasia was performed via inhalation of sevoflurane and additional cervical-dislocation according to the AVMA Guidelines for the Euthanasia of Animals 2013 Edition. All *in vivo* studies were performed in strict accordance with the directive 2010/63/EU on the protection of animals used for scientific purposes and the German animal protection act. Permission for the conduction of the animal experiments was obtained from the committee on animal welfare of the federal state Mecklenburg-Vorpommern, Germany (LALLF M-V; 7221.3–1.1-020/11). All efforts were made to minimize suffering and ensure the highest ethical and human standards. All mouse handling was carefully performed by qualified personnel in accordance to FELASA guidelines.

### *In vivo* infection and determination of bacterial burden

For *in vivo* experiments mice received the respective dose as indicated for each experiment intravenously (i.v.). All mice were monitored for clinical signs and symptoms at least every 8 hours for the duration of the trial. Early endpoint euthanasia to limit pain and distress was employed immediately in case of paraplegia, tetraplegia, severe lethargy, hunching plus ataxia or tremor, or any other condition, which interferes significantly with locomotion, feeding or drinking. Of all mice used for mortality, 13 in total were found dead. The probable cause of death was acute septic shock, which is typical for infections with B. pseudomallei and can occur within a few hours. All remaining mice were euthanized at the end of the experiment. To evaluate the bacterial burden in internal organs, mice were sacrificed on day one or two after infection and their spleens and livers were homogenized and plated onto Ashdown agar plates in appropriate dilutions. Data are presented as total bacterial count per organ.

### IL-10 treatment

Each animal received in total 300 ng murine IL-10 (mIL-10 #5261, Cell Signalling, Germany) intraperitoneally during the experiment: 100 ng in 200 μl PBS were administered one hour prior to infection, additional 100 ng were given at 6 hours and 24 hours after infection, respectively. Controls received 200 μl PBS at each time point. Animals were randomly allocated to a treatment group.

### Generation and cultivation of primary bone-marrow-derived macrophages

Macrophages were generated in a serum-free cell culture system as previously described [23]. Briefly, tibias and femurs were aseptically removed and bone marrow cells were flushed with sterile PBS and then centrifuged at 150 x g for 10 min. Cells were resuspended in RPMI medium containing 5% “Panexin BMM” (PAN Biotech, Germany), 2 ng/ml recombinant murine GM-CSF (PAN Biotech) and 50 μM mercaptoethanol and cultivated for at least 10 days at 37°C and 5% CO_2_. Twenty-four hours prior to infection experiments, mature macrophages were seeded in 48 or 96-well plates as indicated. We could show that the cultivation of stem cells for 10 days in a GM-CSF containing serum-free medium resulted in a homogenous population with very low contamination of lymphoid cells (0.03–0.3%). Macrophages were highly positive for the myeloid markers F4/80, CD11b, MOMA-2 and CD13 (please check reference [23] for further information).

### Invasion- and replication assays

To determine the bactericidal capacity of macrophages, cells were seeded in 48-well plates (~10,000 cells per well) and infected with *B*. *pseudomallei* strain E8 using a MOI as indicated. After infection for 30 min cells were washed twice with PBS and incubated in 100 μg/ml kanamycin containing medium to eliminate remaining extracellular bacteria. To minimize re-infection and extracellular replication the culture medium was replaced by fresh medium containing 100 μg/ml kanamycin six hours after infection. At indicated time points (with time zero taken as 30 min after incubation under antibiotic-containing medium), the number of intracellular CFU was determined as previously described [18]. In some experiments, cells were stimulated with 20 ng/ml murine IL-10 24 hours prior and during incubation.

### Lactate dehydrogenase (LDH) assay

To quantify the extent of cell damage after infection, release of LDH in cell culture supernatants was determined. Macrophages were seeded in 96-well plates (3 × 10^4^ cells/well) and infected with the indicated MOI for 30 min. Cells were washed twice with PBS, and 100 μl of medium containing 125 μg/ml of kanamycin was added to each well to eliminate extracellular bacteria. At the indicated time points, cell culture supernatant was collected, and LDH activity was detected by using a CytoTox-One homogeneous membrane integrity assay (Promega Corp., Madison, USA) according to the manufacturer’s instructions. Briefly, 50 μl of supernatant was added to the kit reagent and incubated for 10 min before stopping solution was added. The fluorescence intensity was measured using a microplate reader (Infinite Type M200 Pro, Tecan; excitation wavelength, 560 nm; emission wavelength, 590 nm).

### Real-time cell status analysis

Macrophages were seeded in 96-well E-plates (40.000 cells/well) and grown for 24 hours at 37°C and 5% CO_2_, before they were infected with *B*. *pseudomallei* (MOI ~5) for 30 min. Cells were further incubated in medium containing 125 μg/ml kanamycin for four days_._ Cellular events were monitored in real-time by measuring the electrical impedance across microelectrodes integrated in the bottom of the E-plates using the xCELLigence system (Roche, Germany). The xCELLigence system calculates changes in impedance as a dimensionless parameter called Cell Index.

### Real-time PCR expression analysis of cytokines

For determining inflammatory parameters, RNA samples were collected from macrophages or spleen tissues at indicated time points. RNA isolation and reverse transcription were performed using standard protocols. qRT-PCR experiments were performed with LightCycler 480 (Roche) and Data was analyzed with the Light Cycler software version 1.5. The gene RPLP0 served as a reference gene for the standardization of the individual PCR. TaqMan PCR probes and gene-specific primer pairs (See S1 File for primer sequences) were generated by Microsynth (Balgach, Switzerland). All assays were performed in duplicate and repeated independently four to six times.

### Statistics

To determine significant differences between groups, either Student’s t-test or two-way Anova test were used as indicated for each experiment. All tests were adjusted for multiple comparisons. Survival curves were compared using the log rank Kaplan-Meier test. Significances were referred as follows: * for p<0.05, ** for p<0.01 and *** for p<0.001. Statistical analyses were performed using GraphPad Prism version 5.

## Results

### *Caspase-6*^*-/-*^ mice are highly susceptible against *B*. *pseudomallei* challenge

**Fig 1:**
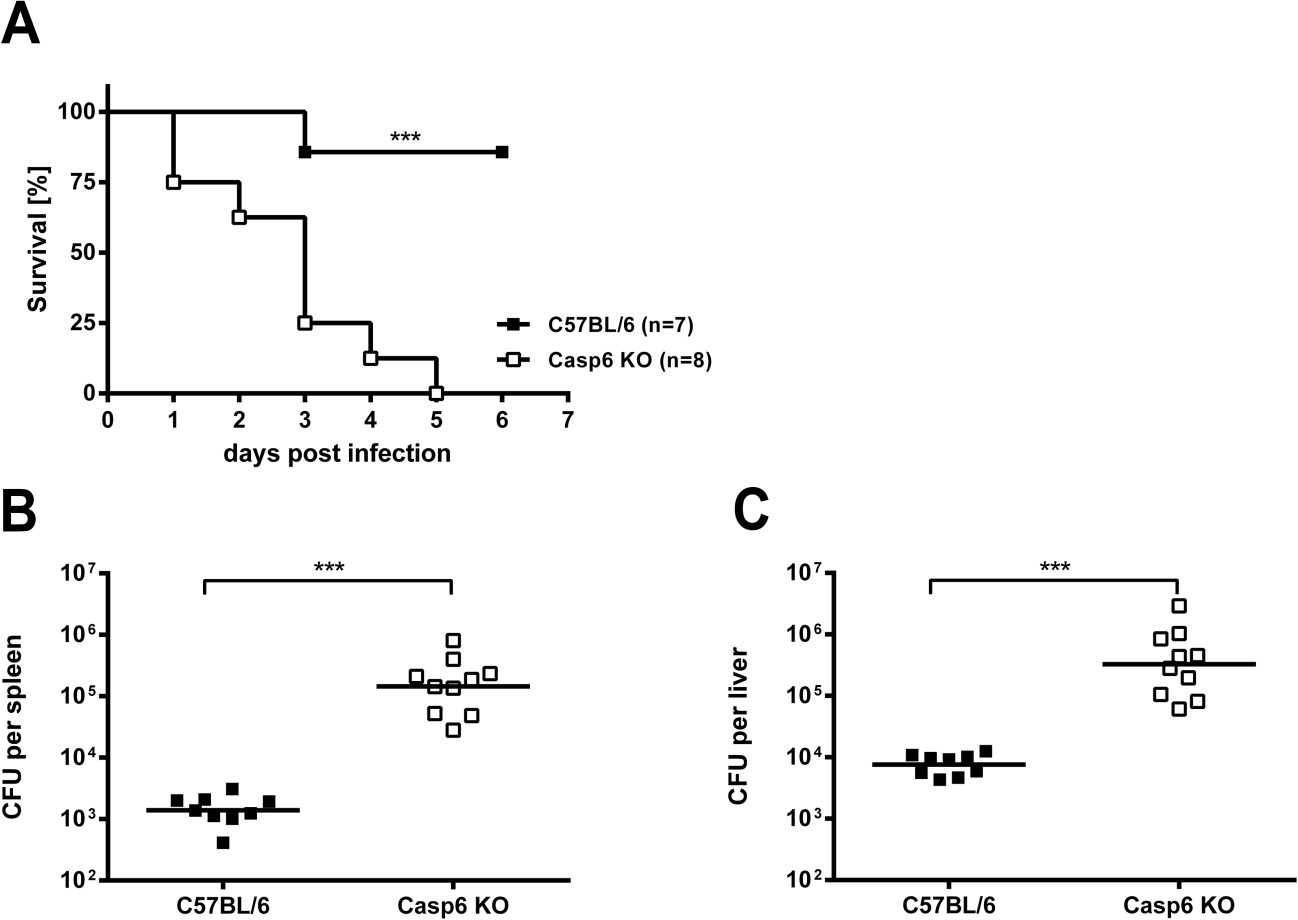
Effect of caspase-6 deficiency on mortality and bacterial burden after infection with *B*. *pseudomallei*. (A) Survival curves of C57BL/6 WT and *caspase6*^*-/-*^ mice after i.v. infection with *B*. *pseudomallei* strain E8 (A, infection dose 2–3 × 10^5^ CFU). Data were analysed using the Kaplan Meier log rank test. (B, C) Bacterial loads in spleens (B) and livers (C) of *B*. *pseudomallei* E8-infected C57BL/6 WT (n = 9) and *caspase6*^*-/-*^ mice (n = 10) (infection dose 5 × 10^4^ CFU) were determined 24 hours after i.v. infection. The data were logarithmized to achieve normal distribution and compared using Student’s t-test. The horizontal line represents the geometric mean of each group. Data were obtained from three independent experiments.

In a first set of experiments we infected C57BL/6 *caspase-6*^*-/-*^ mice i.v. or i.n. with a soil isolate of *B*. *pseudomallei*, strain E8. Mice lacking caspase-6 succumbed significantly earlier than WT animals in both approaches (i.v. infection: Fig 1A, i.n. infection: S1 Fig). In addition, the bacterial loads of spleens and livers were significantly higher in *caspase-6*^*-/-*^ mice 24 hours after i.v. infection with *B*. *pseudomallei* E8 (Fig 1C and 1D). Thus, caspase-6 clearly contributes to *in vivo* resistance against *B*. *pseudomallei* in an early stage of the infection.

### Caspase-6 contributes to the killing activity of *B*. *pseudomallei* infected macrophages

**Fig 2:**
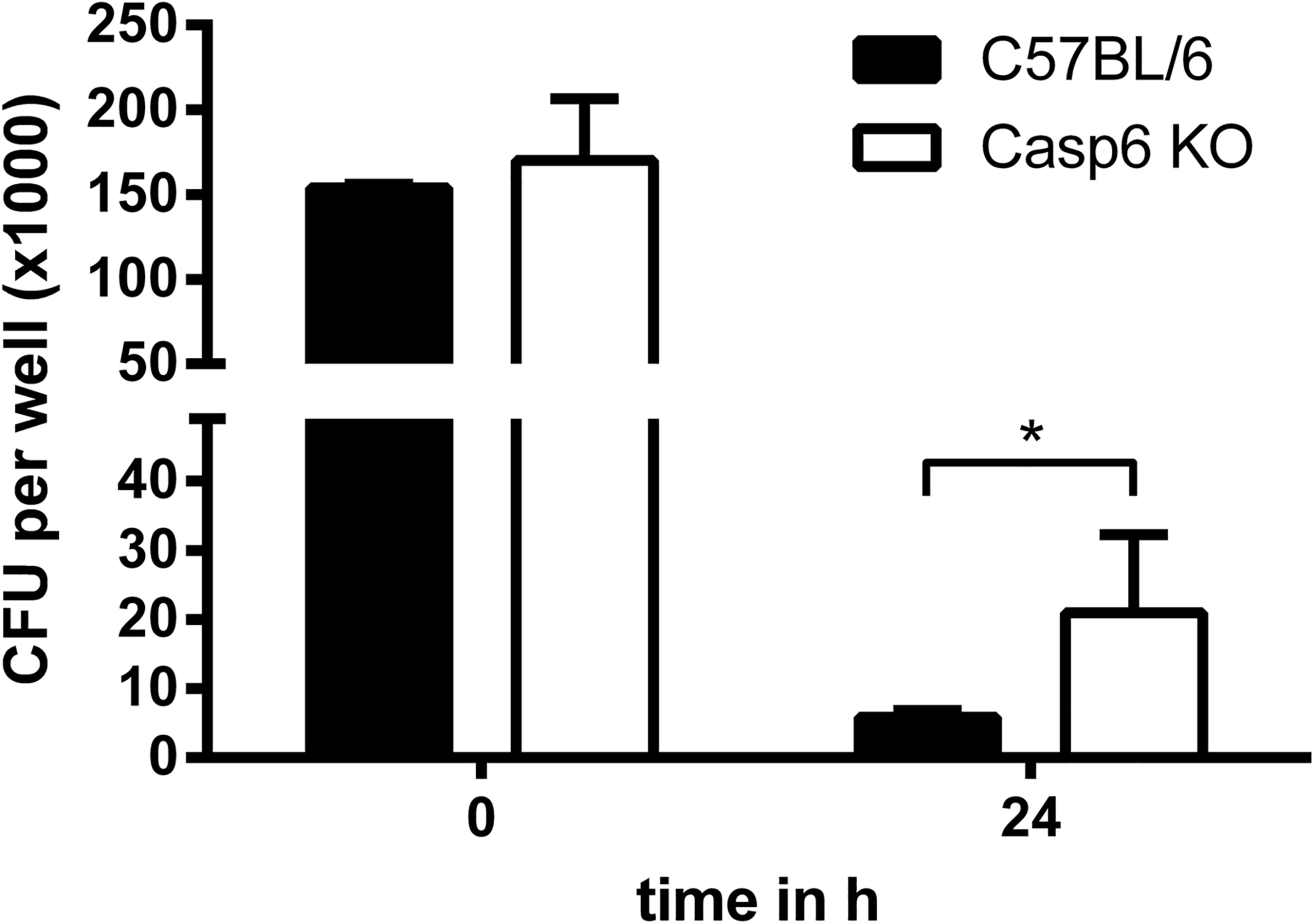
Invasion and replication of *B*. *pseudomallei* in caspase-6 deficient macrophages. Intracellular bacterial burden of C57BL/6-WT and C57BL/6-*caspase6*^*-/-*^ macrophages after infection with *B*. *pseudomallei* strain E8 at an MOI of ~ 25. The CFU data were logarithmized to achieve normal distribution and compared using Student’s t-test. Values are means ± standard deviations from triplicate determinations. The experiment was repeated three times.

In a previous report we provided evidence that macrophages are essential for controlling *B*. *pseudomallei* in the early phase of infection [18]. Since the *in vivo* data led us speculate that caspase-6 confers resistance in an early stage after *B*. *pseudomallei* challenge, we next investigated whether caspase-6 might have an impact on the bactericidal activity of *B*. *pseudomallei* infected macrophages. The uptake of bacteria was not noteworthy different among both macrophage populations (Fig 2). However, although *caspase6*^*-/-*^ macrophages were generally able to reduce the number of intracellular bacteria, the number of the remaining bacteria was still approx. 3-fold higher compared to wild type macrophages 24 hours after infection (Fig 2). Thus, although not dramatically, caspase-6 seems to affect the bactericidal activity of macrophages during *B*. *pseudomallei* infection.

### Caspase-6 does not affect the course of cell death in *B*. *pseudomallei* infected macrophages

**Fig 3:**
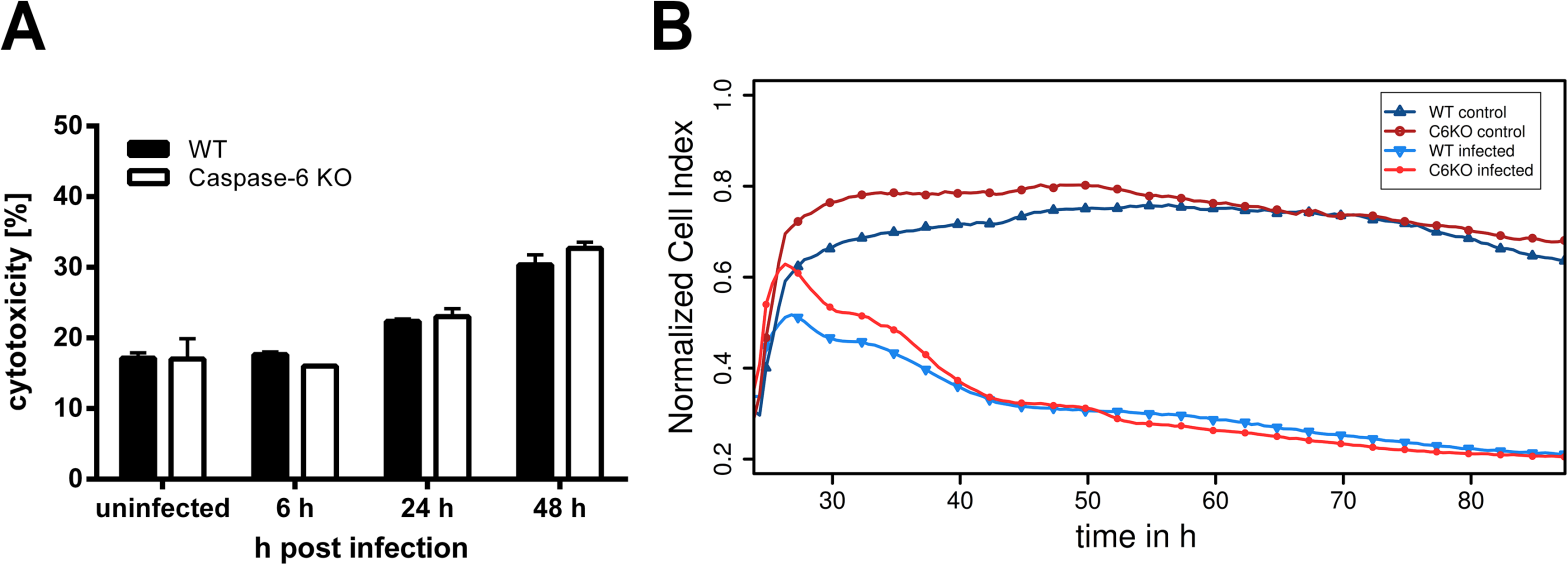
Cell death induction in caspase-6 deficient macrophages after infection with *B*. *pseudomallei*. Course of cell damage in C57BL/6 WT and *caspase6*^*-/-*^ macrophages after infection with *B*. *pseudomallei* strain E8. (A) For the LDH release assay cells were infected with an MOI of ~10. Data were analysed using Student’s *t* test. Values are means ± standard deviations from triplicate determinations. (B) Real-time cell status analysis of C57BL/6 WT (blue, triangles) and *caspase6*^*-/-*^ macrophages (red, circles). Infected cells are represented by brighter lines (top-down triangle, small circle) and received an MOI of ~5. Uninfected controls are represented by darker lines (upright triangle, big circle). The y-axis is a relative scale for the cell status measured by the XCelligence system. The experiments were repeated twice.

Caspases are generally considered to play a role in the induction of cell death in macrophages, as it was shown for the pro-inflammatory caspase-1 after infection with *B*. *pseudomallei* [13,14,24]. Thus, we next examined whether caspase-6 might affect the course of cell death during *B*. *pseudomallei* infection. As shown in Fig 3A, we could not detect any relevant differences in the release of LDH among *caspase6*^*-/-*^ and WT macrophages after *B*. *pseudomallei* infection over a period of 48 h. In addition, we used another system by measuring the electrical impedance that correlates with the vitality of the cells. However, with this approach we were also not able to detect remarkable differences in the course of cell death among the cell populations (Fig 3B). This is in agreement with another report describing that caspase-6 activation is not necessarily associated with the induction of cell death [25]. The present data indicate that caspase-6 dependent mechanisms do not seem to play a role for cell death induction in *B*. *pseudomallei* infected macrophages.

### Lack of caspase-6 is associated with an early increased expression of IL-1β and IL-10 after infection with *B*. *pseudomallei*

**Fig 4:**
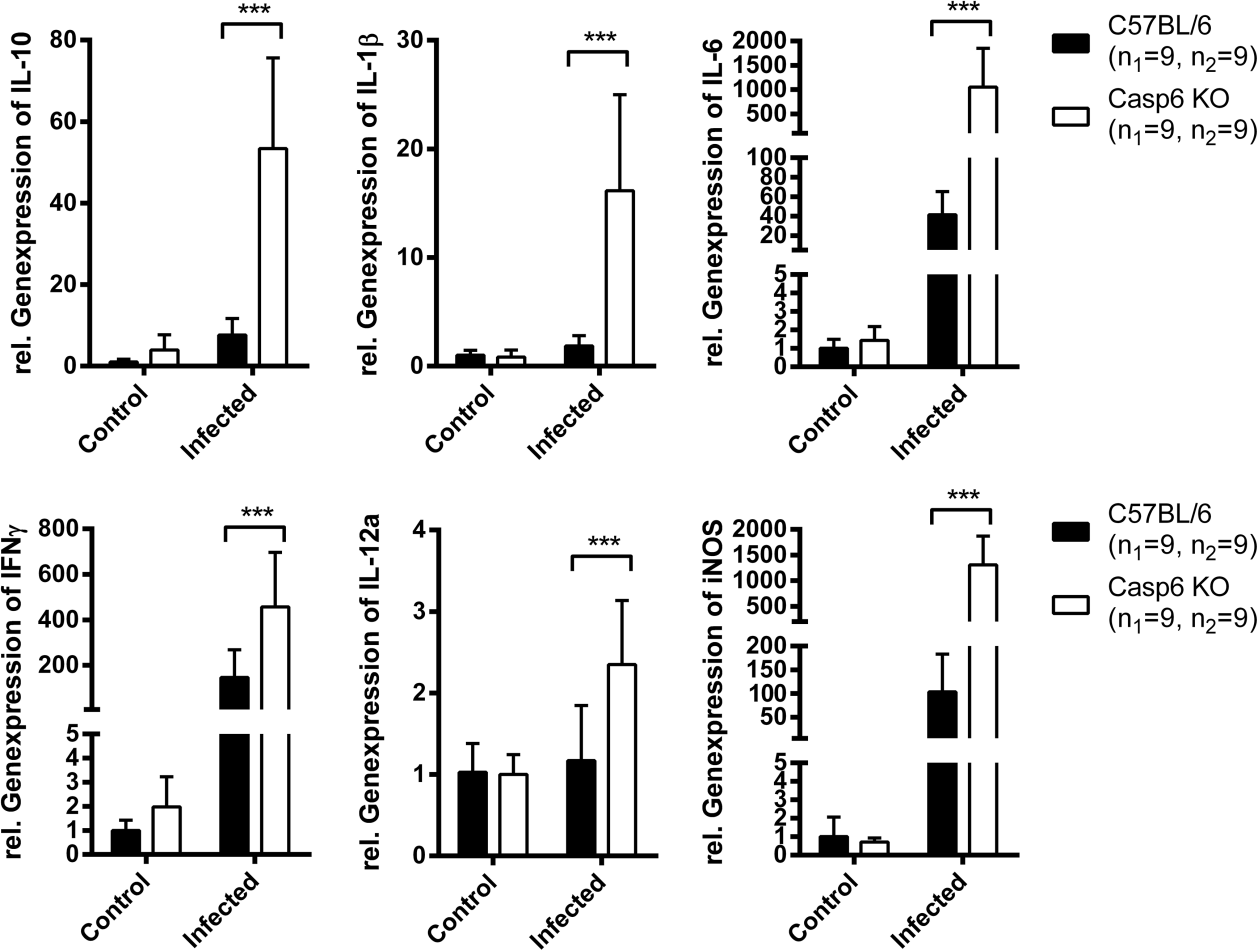
Cytokine expression in caspase-6 deficient mice 24 hours after infection with *B*. *pseudomallei*. Quantitative real-time PCR analysis of inflammatory parameters in the spleens of C57BL/6 WT and *caspase6*^*-/-*^ mice. Mice were infected with 5 × 10^4^ CFU of *B*. *pseudomallei* strain E8 i.v. for 24 h. Uninfected control animals received PBS. Each of the 4 groups contained 9 mice from 3 replicates (n = 36). Data were analysed using Student’s *t* test. Values are means ± standard deviations from three independent experiments.

**Fig 5:**
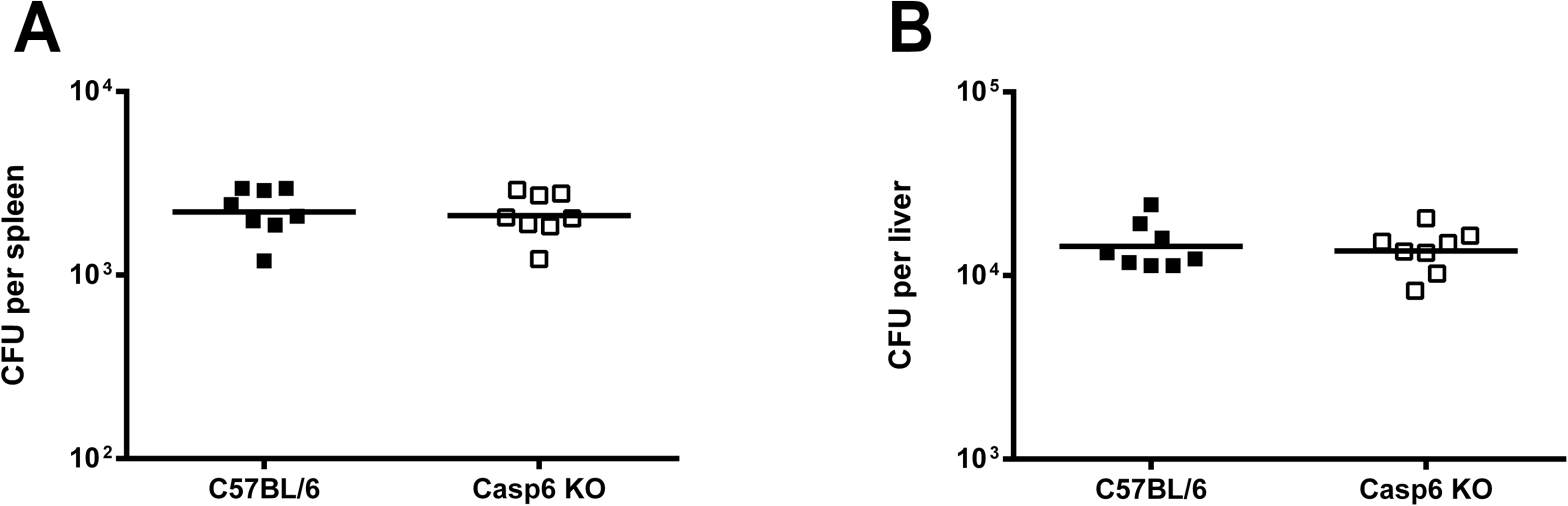
Bacterial burden in caspase-6 deficient mice 6 hours after infection with *B*. *pseudomallei*. (A, B) Bacterial loads in spleens (A) and livers (B) of *B*. *pseudomallei* strain E8-infected C57BL/6 WT (n = 8) and *caspase6*^*-/-*^ mice (n = 8) were determined 6 hours after i.v. infection with 5 × 10^4^ CFU *B*. *pseudomallei* strain E8. The data were logarithmized to achieve normal distribution and compared using Student’s t-test. The horizontal line represents the geometric mean of each group. Data were obtained from two independent experiments.

**Fig 6:**
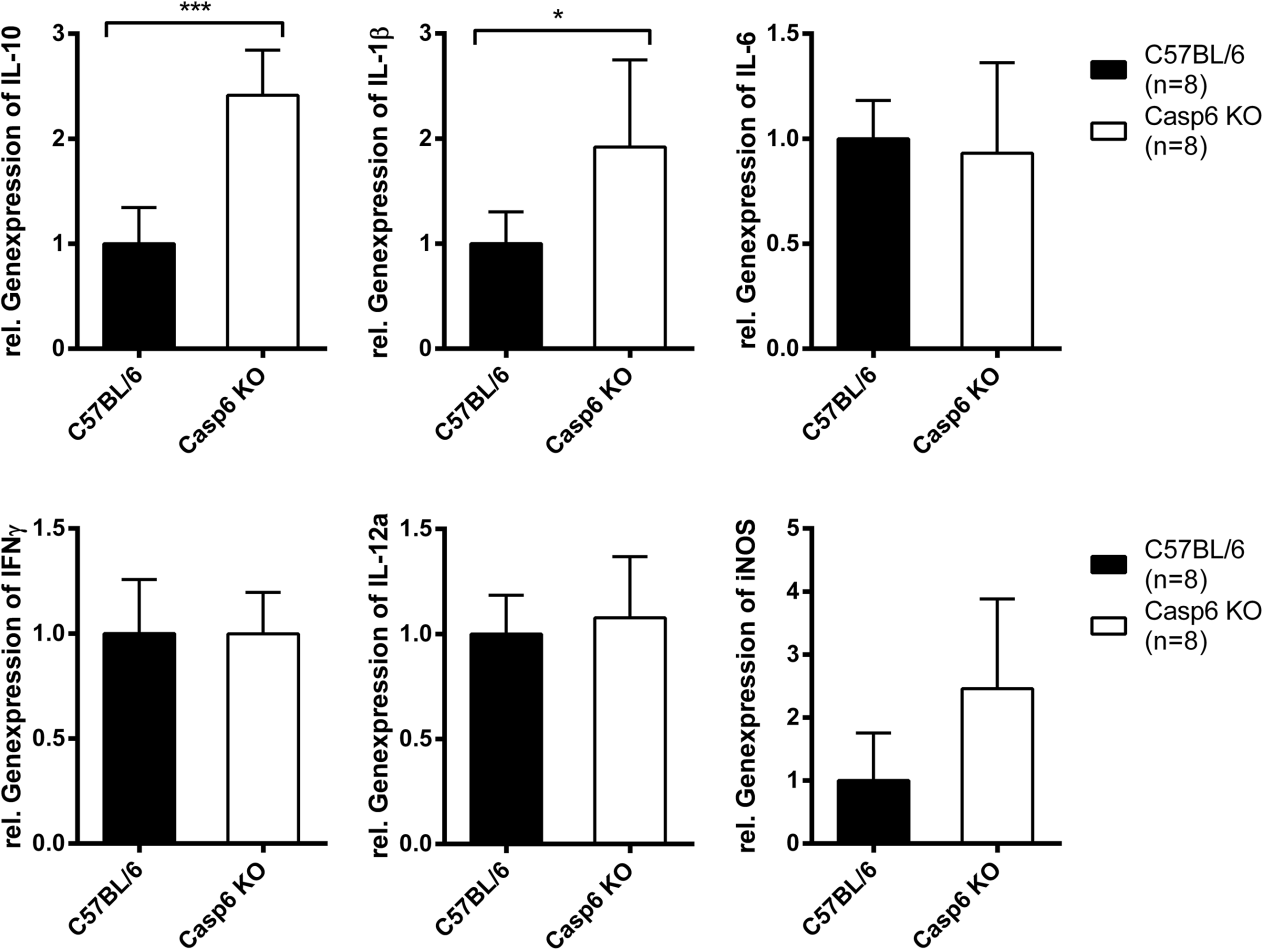
Cytokine expression in caspase-6 deficient mice 6 hours after infection with *B*. *pseudomallei*. Quantitative real-time PCR analysis of inflammatory parameters in the spleens of C57BL/6 WT (n = 8) and *caspase6*^*-/-*^ mice (n = 8) 6 hours after infection with 5 × 10^4^ CFU *B*. *pseudomallei* strain E8. Data were analysed using Student’s *t* test. Values are means ± standard deviations from two independent experiments.

Having shown that caspase-6 plays an important role during the early phase of systemic *B*. *pseudomallei* infection and affected to some degree the bactericidal capacity of macrophages, we assumed that caspase-6 might modulate innate immune functions. We therefore examined the expression of various inflammatory cytokines in the spleens of i.v. infected animals. We chose the i.v. infection route to ensure a constant distribution of the bacteria in internal organs to study innate immune effects in the very early phase of infection. Not unexpected, the expression of various inflammatory parameters such as IFN-γ, IL-6, IL-12, TNF-α, iNOS, IL-1β and IL-10 was significantly increased in spleens of *caspase6*^*-/-*^ mice at 24 hours after infection (Fig 4). However, this was likely due to the increased bacterial burden in these mice compared to WT animals at that time point (Fig 1C). Thus, we next investigated the expression of cytokines at 6 hours after infection and at a time, when the bacterial loads of *caspase6*^*-/-*^ and WT mice were not significantly different in the spleens and livers (Fig 5A and 5B). Among the cytokines tested, IL-1β and IL-10 proved to be significantly higher expressed in spleens from *caspase6*^*-/-*^ mice at 6 hours after infection with *B*. *pseudomallei* (Fig 6). Thus, caspase-6 seems to affect the expression of IL-10 and IL-1β in a very early stage after infection.

### IL-10 is deleterious in mice after *B*. *pseudomallei* infection

**Fig 7:**
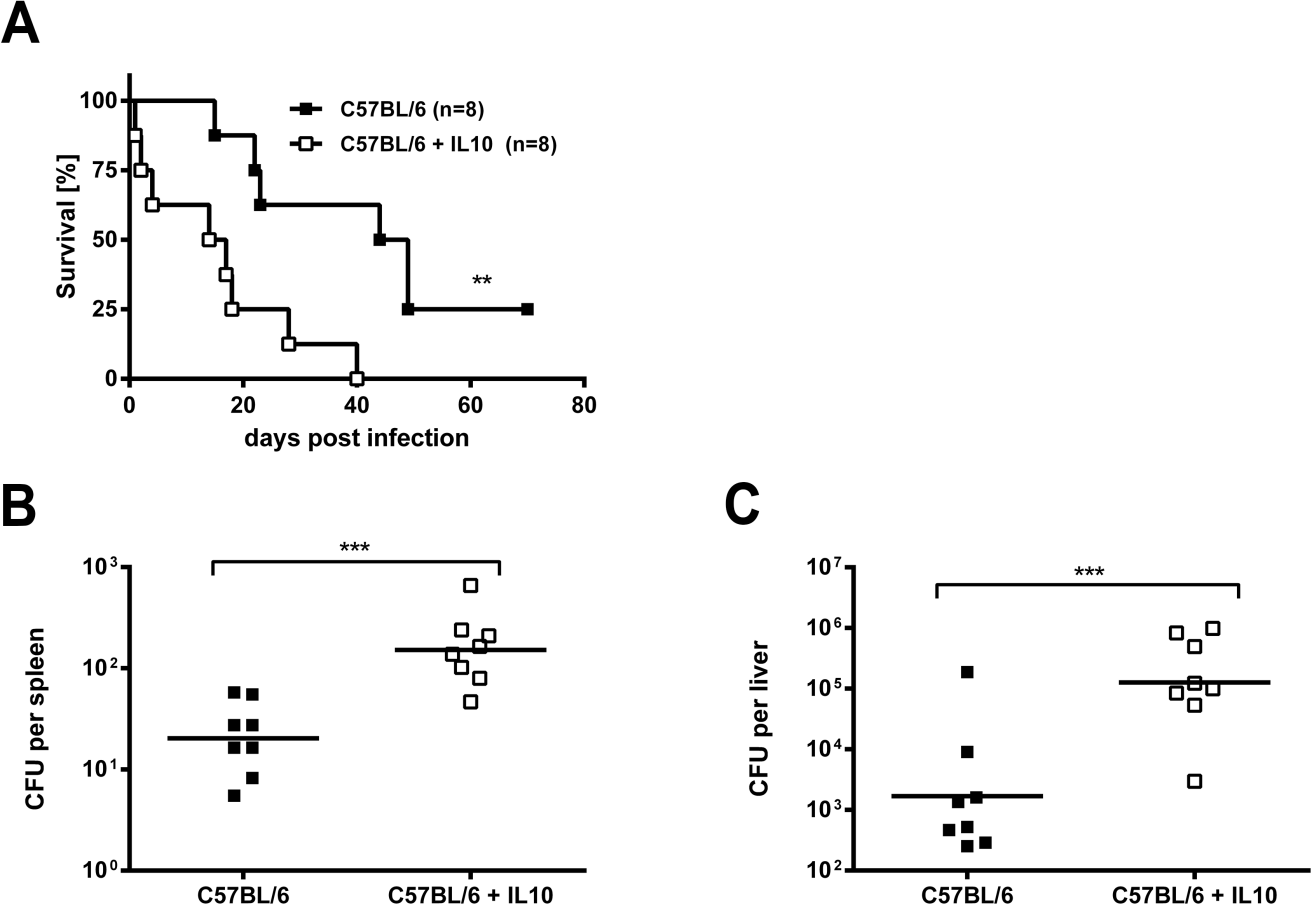
Effect of IL-10 treatment *in vivo* during *B*. *pseudomallei* infection. (A) Survival curves of IL-10-treated and sham-treated C57BL/6 WT mice after i.v. infection with ~4 × 10^5^ CFU of *B*. *pseudomallei* strain E8. Data were analysed using the Kaplan Meier log rank test. (B, C) Bacterial loads in spleens (B) and livers (C) of IL-10-treated (n = 8) and sham-treated C57BL/6 WT mice (n = 8) were determined 48 hours after i.v. infection with ~10^5^ CFU *B*. *pseudomallei* strain E8. The data were logarithmized to achieve normal distribution and compared using Student’s t-test. The horizontal line represents the geometric mean of each group. The data were obtained from two independent experiments.

Since IL-10 and IL-1β were up-regulated at early time points after *B*. *pseudomallei* infection in the absence of caspase-6, we next evaluated the impact of these cytokines in *B*. *pseudomallei* infection. A previous study could already show that IL-1β production had detrimental effects in *B*. *pseudomallei* infected mice by using IL-1RI-deficient mice or WT mice treated with IL-1β [22]. Furthermore, IL-10 production was associated with fatal outcomes in melioidosis patients [26]. However, a direct role for IL-10 in *B*. *pseudomallei* infection and *in vivo* resistance has not yet been addressed. Thus, we tested whether IL-10 might be associated with an impaired outcome after *B*. *pseudomallei* challenge. For this purpose we treated C57BL/6 WT mice with endogenous IL-10 prior to infection. As shown in Fig 7A, IL-10 treated animals showed significantly impaired survival compared to control mice. In addition, the bacterial load in spleens and livers from IL-10 treated animals were significantly higher at 48 hours after infection (Fig 7B and 7C). These data clearly show that an elevated IL-10 level has deleterious effects during *B*. *pseudomallei* infection in mice.

### IL-10 impairs the bactericidal activity of *B*. *pseudomallei* infected macrophages

**Fig 8:**
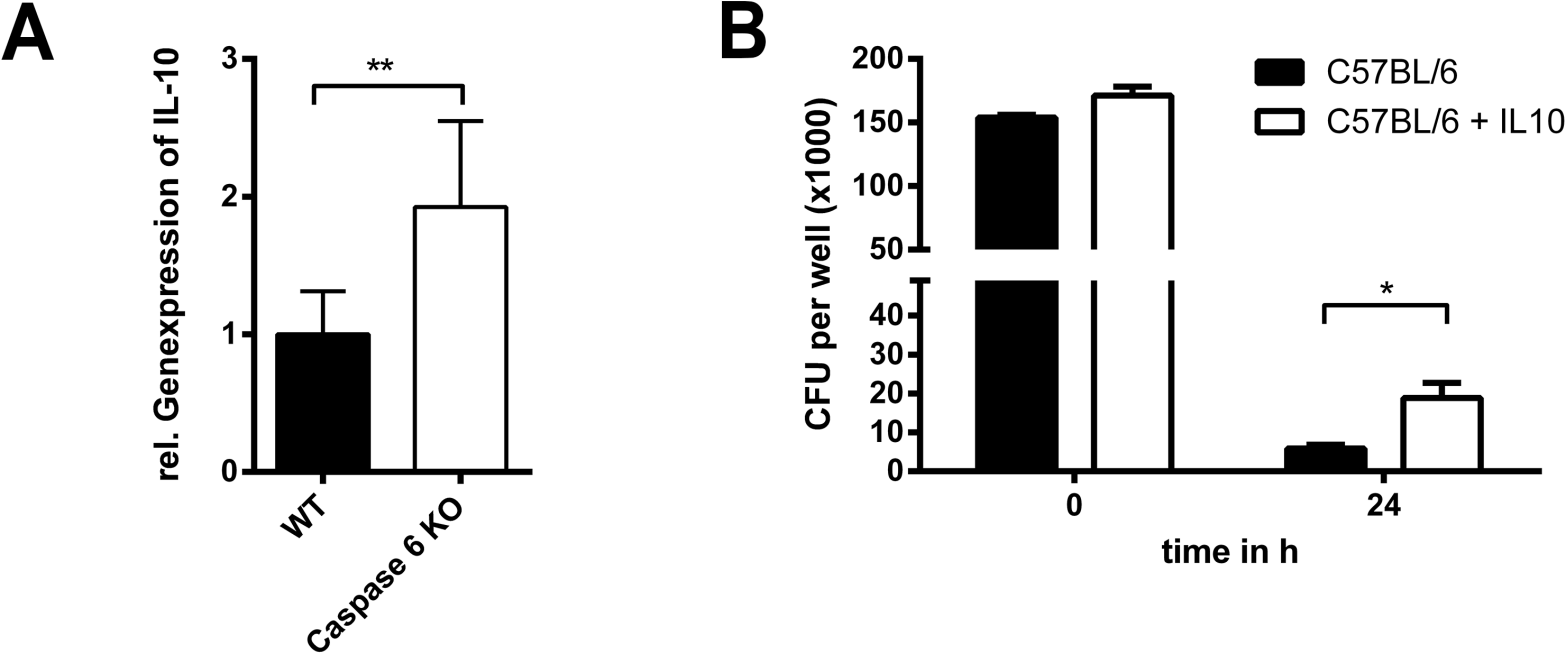
Effect of IL-10 in macrophages during *B*. *pseudomallei* infection. (A) Quantitative real-time PCR analysis of IL-10 expression in macrophages from C57BL/6 WT and *caspase6*^*-/-*^ mice 6 h after infection with *B*. *pseudomallei* strain E8 (MOI ~ 50). The data were logarithmized to achieve normal distribution and compared using Student’s *t*-test. Values are means ± standard deviations from three independent experiments. (B) Intracellular bacterial burden of IL-10-treated and non-treated C57BL/6 macrophages after infection with *B*. *pseudomallei* strain E8 (MOI ~ 25). Data were analysed using Student’s *t*-test. Values are means ± standard deviations from triplicate determinations. The experiment was repeated three times.

Having shown that IL-10 is detrimental during *B*. *pseudomallei* infection, we evaluated whether macrophages were influenced in their bactericidal activity against *B*. *pseudomallei* in the presence of IL-10. First, we showed that IL-10 expression was influenced by caspase-6 since absence of caspase-6 was associated with significant higher expression of IL-10 (Fig 8A). As depicted in Fig 8B, there was no significant difference in the uptake of bacteria in macrophages in the presence of IL-10. Similar to what we found in *caspase6*^*-/-*^ macrophages (Fig 2), there were slightly higher numbers of remaining intracellular bacteria in IL-10 treated macrophages 24 hours after infection compared to cells that were left untreated (Fig 8B). Thus, IL-10 seems to negatively affect the bactericidal potential of macrophages. This might contribute to the increased susceptibility of *caspase6*^*-/-*^ mice in very early stages after *B*. *pseudomallei* challenge.

## Discussion

Caspase-6 has yet received thorough attention in the field of neurodegenerative diseases to understand the pathogenesis of Huntingon’s and Alzheimer’s diseases for achieving novel therapeutic strategies [3,4,27]. Beside this, a function for caspase-6 has occasionally been addressed in the context of various cell death pathways such as apoptosis, necroptosis and autophagy [6,28] and the differentiation of B-cells [29]. It is also known that caspase-6 is a substrate for several transcription factors such as NF-κB, AP-2α, CREB-binding proteins and IRAK-M [7,30–32]. But although caspase-6 is known to have a function in controlling immunological processes, its role in infectious diseases has rarely been addressed. Some reports describe that caspase-6 is activated during infection with various pathogens in vitro [33–35]. To our knowledge there is only one report available in that the role of caspase-6 was addressed *in vivo* in a cecal lesion induced peritonitis and sepsis model [7]. However, this approach does not include a standardised infection protocol with the application of defined infection doses. Moreover, the individual gut flora of the animals might contain hundreds of different species. Thus, whether caspase-6 plays a role for determining resistance against microbial pathogens has not been addressed in any defined *in vivo* infection model yet.

The data presented in this report clearly show that absence of caspase-6 rendered C57BL/6 mice highly susceptible against the Gram-negative rod *B*. *pseudomallei*. A similar phenomenon was observed in the absence of caspase-1/11 in a previous study [13]. Whereas the increased susceptibility in *caspase-1/11*^-/-^ mice was clearly associated with reduced expression of protective IFN-γ [13,22], we found *caspase-6*^*-/-*^ mice even to exhibit increased mRNA expression levels of IFN-γ at 24 hours after infection when the bacterial load was approx. 100-fold higher in the knockout animals compared to the WT animals. The expression of other pro-inflammatory cytokines such as IL-12 and TNF-α was also significant increased. However, these differences were likely caused by secondary effects as a result of the different bacterial load. For this reason we checked the mRNA cytokine expression levels at a time when the bacterial load was comparable among WT and *caspase-6*^*-/-*^ mice (6 h post infection). At this time point the IFN-γ expression did not differ among the animal groups. However, we found that *caspase-6*^*-/-*^ mice exhibited significantly increased mRNA expression levels of IL-10 and IL-1β at that time point.

A previous report from Ceballos-Olvera and colleagues could convincingly show that caspase-1 dependent IL-1β expression had rather detrimental effects on the host, while caspase-1 dependent IL-18 expression was crucial for the host’s resistance, primarily due to IL-18 dependent IFN-γ production. Thus, the effect of IL-18 dependent IFN-γ expression is likely to compensate the damaging effects caused by IL-1β and in summary led to a caspase-1 dependent protection. The increased mRNA expression levels of deleterious IL-1β in *caspase-6*^*-/-*^ mice is a possible mechanism to contribute to the increased susceptibility in the absence of caspase-6. In addition, *caspase-6*^-/-^ mice exhibited increased mRNA expression of IL-10, another cytokine that had detrimental effects during *B*. *pseudomallei* infection. Several reports have already described that increased expression of IL-10 was associated with a fatal outcome in melioidosis. One study described IL-10 to serve as an independent predictor of mortality [26]. Moreover, patients with a mutated TLR5, that led to a reduced flagellin-activated immune response, had lower levels of IL-10, and this correlated with a better outcome after *B*. *pseudomallei* infection compared with patients harbouring non-mutated alleles [36]. In the present study we provided direct evidence that IL-10 was detrimental during infection with *B*. *pseudomallei* since IL-10 treated mice succumbed earlier than the controls and exhibited significant higher bacterial loads at 48 hours after infection. Moreover, the bactericidal activity of macrophages was slightly impaired in the presence of IL-10.

IL-10 is an important negative immune regulator preventing the host’s tissue from damage caused by pro-inflammatory processes. On the other hand it is known that IL-10 production can reduce the host’s ability to clear infections with various pathogens such as *Mycobacterium ssp*., *Listeria monocytogenes* and *Leishmania major* [37]. It is known that IL-10 can inhibit the macrophage microbicidal activity by blocking the phagolysosome maturation [38,39]. In this context it was shown that IL-10 gene repression increased the antimicrobacterial activity of macrophages [40]. Thus, it is likely that IL-10 treated macrophages have difficulties in controlling infection with the intracellular bacterium *B*. *pseudomallei* due to insufficient phagolysosome function.

Together, this is the first report addressing the role of caspase-6 for *in vivo* resistance in a defined infection model. We found caspase-6 to be highly essential for conferring resistance against the bacterial pathogen *B*. *pseudomallei*. Absence of caspase-6 was associated with significantly increased mRNA expression levels of two cytokines in a very early stage after infection, the anti-inflammatory IL-10 and the pro-inflammatory IL-1β, that are marker of poor disease outcome. Caspase-6 seems therefore to have a pivotal role in the repression of detrimental cytokines during the course of systemic melioidosis. Thus, caspase-6 might contribute to achieve a well-balanced immune response that can efficiently eliminate the pathogen by preventing unnecessary damage from the host. Future studies are needed to unravel further underlying mechanisms that are involved in the caspase-6 dependent cytokine repression to gain a better understanding about the host’s strategies to efficiently combat microbial pathogens. This might also offer the opportunity for developing novel therapeutic strategies.

## Supporting information

S1 Fig Effect of caspase-6 deficiency on mortality after *intranasal* infection with *B*. *pseudomallei* strain E8. Survival curves of C57BL/6 WT and *caspase6*^*-/-*^ mice after i.n. infection with *B*. *pseudomallei* strain E8 (infection dose 300–400 CFU) Data were analysed using the Kaplan Meier log rank test.
(TIF)

S1 File Primer used for quantitative real-time PCR analysis. (DOCX)
