# Supplementary figures and images for: Caspase-6 mediates resistance against Burkholderia pseudomallei infection and influences the expression of detrimental cytokines
Source: PLoS One. 2017 Jul 7;12(7):e0180203. doi: 10.1371/journal.pone.0180203 (PMC5501493; doi:10.1371/journal.pone.0180203)

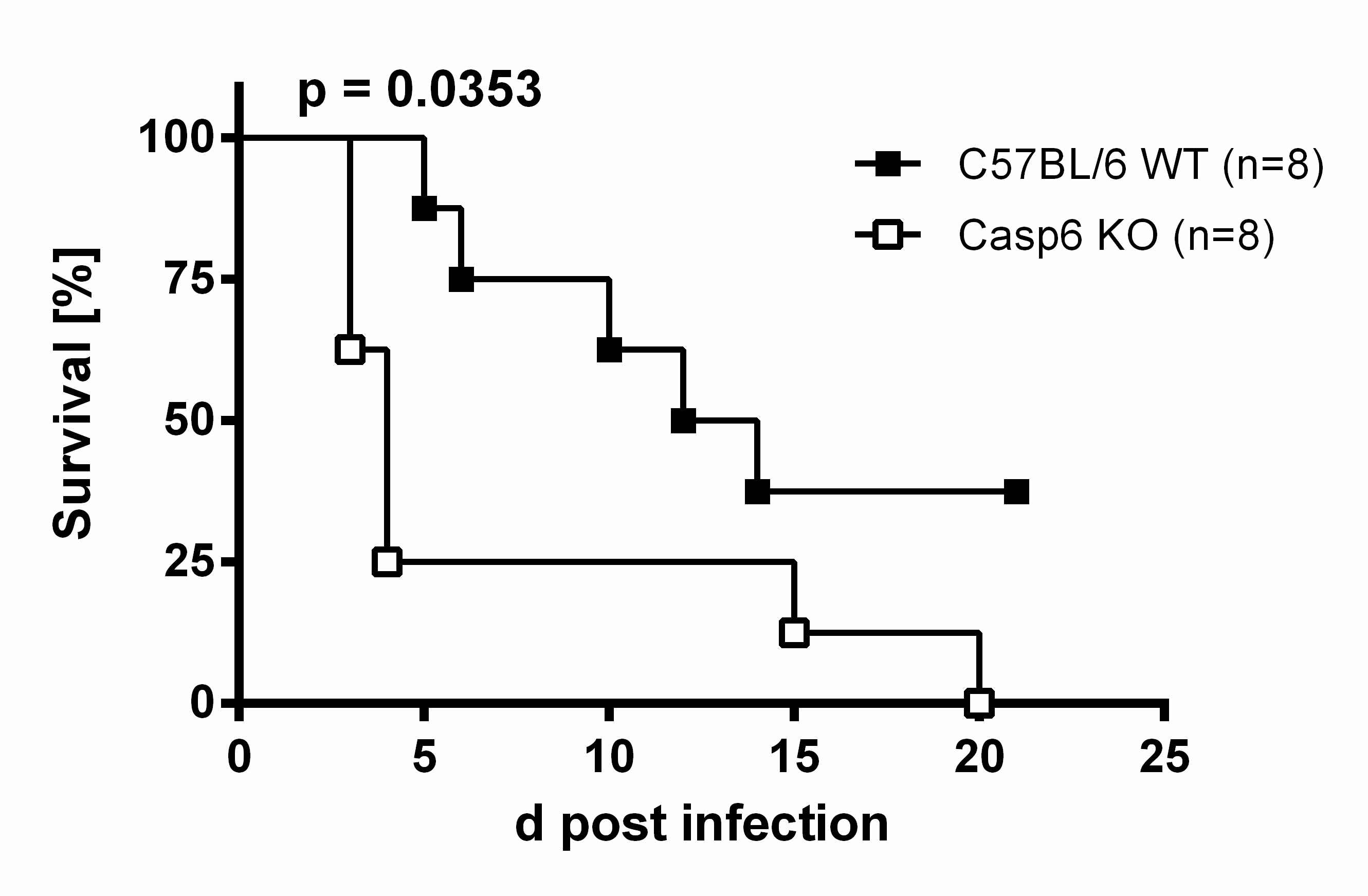

Supplement: S1 Fig — Survival curves of C57BL/6 WT and caspase6-/- mice after i.n. infection with B. pseudomallei strain E8 (infection dose 300–400 CFU) Data were analysed using the Kaplan Meier log rank test. (TIF) [file pone.0180203.s001.tif]
