# Supplementary material for: Caspase-6 mediates resistance against Burkholderia pseudomallei infection and influences the expression of detrimental cytokines
Source: PLoS One. 2017 Jul 7;12(7):e0180203. doi: 10.1371/journal.pone.0180203 (PMC5501493; doi:10.1371/journal.pone.0180203)
Supplement: S1 File — (DOCX) [file pone.0180203.s002.docx]

| Target | Type | Size | Sequenz |
| --- | --- | --- | --- |
| RPLP0 | for | 649 | 5’ TGA GAT TCG GGA TAT GCT GTT GG 3’ |
|  | rev |  | 5’ TGT TCT GAG CTG GCA CAG TG 3’ |
|  | probe |  | 5’ FAM CCT CAC ACG GGG CGA TGG CAC C TAMRA 3’ |
| IL-10 | for | 247 | 5’ TGC AGG ACT TTA AGG GTT A 3’ |
|  | rev |  | 5’ CCT TGG TCT TGG AGC TTA 3’ |
|  | probe |  | 5’ FAM CAC TCT TCA CCT GCT CCA CTG TAMRA 3’ |
| IL-1b | for | 97 | 5’ CAG CAG CAC ATC AAC AAG AGC 3’ |
|  | rev |  | 5’ GGA AGG TCC ACG GGA AAG AC 3’ |
|  | probe |  | 5’ FAM TGC CAC AGC TTC TCC ACA GCC ACA TAMRA 3’ |
| IL-6 | for | 167 | 5’ GCC AGA GTC CTT CAG AGA GAT AC 3’ |
|  | rev |  | 5’ AAG ATG AAT TGG ATG GTC TTG GTC 3’ |
|  | probe |  | 5’ FAM AGC CAC TCC TTC TGT GAC TCC AGC T TAMRA 3’ |
| IL-12a | for | 131 | 5’ CAG CAC ATT GAA GAC CTG 3’ |
|  | rev |  | 5’ ACA GGG TCA TCA TCA AAG 3’ |
|  | probe |  | 5’ FAM CAC TGG AAC TAC ACA AGA ACG AGA G TAMRA 3’ |
| IFN-g | for | 164 | 5’ CCA AGT TTG AGG TCA ACA 3’ |
|  | rev |  | 5’ CTG GCA GAA TTA TTC TTA TTG 3‘ |
|  | probe |  | 5’ FAM CGA ATC AGC GAC TCC TT TAMRA 3’ |
| iNOS | for | 139 | 5’ AAG GGA CTG AGC TGT TAG AGA 3‘ |
|  | rev |  | 5’ CAA GCC ATG TCT GAG ACT TTG C 3’ |
|  | probe |  | 5’ FAM AGG CTC CTC ACG CTT GGG TCT TGT TAMRA 3’ |

**S2 File. Primer used for quantitative real-time PCR analysis**
